# Uncovering the Connectivity Logic of the Ventral Tegmental Area

**DOI:** 10.3389/fncir.2021.799688

**Published:** 2022-01-28

**Authors:** Pieter Derdeyn, May Hui, Desiree Macchia, Kevin T. Beier

**Affiliations:** ^1^Program in Mathematical, Computational, and Systems Biology, University of California, Irvine, Irvine, CA, United States; ^2^Department of Physiology and Biophysics, University of California, Irvine, Irvine, CA, United States; ^3^Department of Neurobiology and Behavior, University of California, Irvine, Irvine, CA, United States; ^4^Department of Biomedical Engineering, University of California, Irvine, Irvine, CA, United States; ^5^Department of Pharmaceutical Sciences, University of California, Irvine, Irvine, CA, United States; ^6^Center for the Neurobiology of Learning and Memory, University of California, Irvine, Irvine, CA, United States

**Keywords:** VTA (ventral tegmental area), rabies, circuit mapping, dopamine, inputs and outputs, high dimension datasets, spatial patterning

## Abstract

Decades of research have revealed the remarkable complexity of the midbrain dopamine (DA) system, which comprises cells principally located in the ventral tegmental area (VTA) and substantia nigra pars compacta (SNc). Neither homogenous nor serving a singular function, the midbrain DA system is instead composed of distinct cell populations that (1) receive different sets of inputs, (2) project to separate forebrain sites, and (3) are characterized by unique transcriptional and physiological signatures. To appreciate how these differences relate to circuit function, we first need to understand the anatomical connectivity of unique DA pathways and how this connectivity relates to DA-dependent motivated behavior. We and others have provided detailed maps of the input-output relationships of several subpopulations of midbrain DA cells and explored the roles of these different cell populations in directing behavioral output. In this study, we analyze VTA inputs and outputs as a high dimensional dataset (10 outputs, 22 inputs), deploying computational techniques well-suited to finding interpretable patterns in such data. In addition to reinforcing our previous conclusion that the connectivity in the VTA is dependent on spatial organization, our analysis also uncovered a set of inputs elevated onto each projection-defined VTA^DA^ cell type. For example, VTA^DA^→NAcLat cells receive preferential innervation from inputs in the basal ganglia, while VTA^DA^→Amygdala cells preferentially receive inputs from populations sending a distributed input across the VTA, which happen to be regions associated with the brain’s stress circuitry. In addition, VTA^DA^→NAcMed cells receive ventromedially biased inputs including from the preoptic area, ventral pallidum, and laterodorsal tegmentum, while VTA^DA^→mPFC cells are defined by dominant inputs from the habenula and dorsal raphe. We also go on to show that the biased input logic to the VTA^DA^ cells can be recapitulated using projection architecture in the ventral midbrain, reinforcing our finding that most input differences identified using rabies-based (RABV) circuit mapping reflect projection archetypes within the VTA.

## Introduction

The VTA plays a central role in a variety of both adaptive and pathological motivated behaviors, principally through cells that release the neurotransmitter DA (Morales and Margolis, 2017). These cells direct motivated behaviors by release of DA into downstream brain structures such as the nucleus accumbens (NAc), dorsal striatum (DStr), and medial prefrontal cortex (mPFC) (Beier et al., 2015). Activation of DA cells as a population is highly reinforcing, as animals will robustly self-administer stimulation of DA neurons (Olds and Milner, 1954). DA cells have also been implicated in reward-prediction error (RPE), or the difference between the received and anticipated value of an outcome (Schultz, 1998). While much of the data fit the RPE model, some do not. For example, an aversive stressful experience or a painful stimulus such as a foot pinch triggers DA release into forebrain structures (Navratilova et al., 2015). One recent study suggested that physiological DA release in the NAc only relates to outcomes predicted by RPE within a limited number of scenarios and instead broadly signals perceived salience (Kutlu et al., 2021). Other studies pointed to the existence of subsets of DA cells that not only project to different forebrain sites, but also have unique transcriptional, electrophysiological, and response properties to various stimuli (Lammel et al., 2008, 2011; Kim et al., 2016). We now know that the VTA is comprised of heterogenous cell types: DA cells comprise roughly 50% of VTA cells in the rat, fewer than the >70% previously estimated (Margolis et al., 2006); another ∼40% of cells in the VTA are GABAergic. Many of these GABAergic cells inhibit VTA^DA^ neurons, and their activation has the opposite effect of DA cell stimulation (Bouarab et al., 2019). In addition to locally inhibiting DA cells, VTA*^GABA^* neurons also project to a variety of forebrain sites, including the NAc and lateral habenula (LHb). Many VTA*^GABA^* cells can also co-transmit glutamate (Root et al., 2014). Additionally, many NAc-projecting midbrain DA cells co-transmit glutamate, and some can also synthesize and transmit GABA through a non-canonical pathway (Tritsch et al., 2012; Kim et al., 2015). This complexity makes it difficult to definitively disentangle the roles that various cells play in adaptive and maladaptive behaviors.

To date, DA cells have typically been differentiated based on output site. For example, Lammel et al. (2008) injected fluorescent microspheres into different forebrain sites and showed that the DA cells in the midbrain that took up the microspheres were largely distinct, as these cell populations differed in their expression of dopamine transporter, DAT, and in their electrophysiological properties. They later showed that these cells were differentially modulated by experience, as the synapses onto some cells and not others were modulated by either a cocaine (rewarding) or formalin (aversive) experience (Lammel et al., 2011). These results suggested that these cells are integrated into separate circuits that are differentially involved in either reward or aversion learning. The same investigators then showed that VTA^DA^ cells projecting to the NAc preferentially received inputs from the laterodorsal tegmentum (LDT) and signaled reward, whereas VTA^DA^ cells projecting to the mPFC preferentially received inputs from the LHb and signaled aversion (Lammel et al., 2012). These studies provided a simplified framework through which VTA^DA^ neurons could encode both reward and aversion-related signals through separate forebrain projections. Subsequent studies have largely supported this framework, with some modifications. We, therefore, wanted to explore the global anatomical organization of these cells and examine how connectivity logic may help to explain the roles different DA cells play in behavior. As midbrain DA cells have been shown to receive direct monosynaptic inputs from over 100 anatomically defined brain regions (Watabe-Uchida et al., 2012), our goal has been to create comprehensive input-output connectivity maps of discrete DA populations to compare the inputs and outputs of these cells.

To unambiguously define input-output relationships of midbrain DA cells, we developed an intersectional viral-genetic method to tag cells defined by both gene expression and output site, termed cell type-specific Tracing the Relationship of Inputs and Outputs (cTRIO) (Beier et al., 2015; Schwarz et al., 2015). In our initial study, we characterized the input-output relationships of VTA^DA^ cells projecting to the nucleus accumbens (NAcMed and NAcLat), medial prefrontal cortex (mPFC), and Amygdala (Beier et al., 2015). cTRIO revealed separate sub-circuits centered on midbrain DA cells that had biased inputs and discrete outputs. We then performed a more detailed characterization of the connectivity relationships of these populations (Beier et al., 2019), finding that the spatial location of starter cells in the VTA was the main determinant of the inputs that each population received while the neurotransmitters that the cells released did not strongly influence input patterns. However, relating the center of mass (COM) of “starter” neurons that initiate RABV tracing to input fraction using a simple linear regression only explained significant variance for about half of the input sites examined, suggesting that this level of analysis was not sufficient to explain the full complexity of input patterning to the VTA. Quantitative techniques have been adopted in other fields to reveal patterns in high dimensional data. In this study we aim to introduce such techniques to neural circuit mapping. We revisit previously published datasets describing the inputs and outputs of VTA^DA^ cells and find new patterns and rules underlying their connectivity.

## Results

### VTA^DA^ Neurons Segregate by Projection Condition With Characteristic Output Patterns

Lammel et al. (2008) first used retrobead injections into different forebrain regions in the mouse to show that DA cells projecting to different forebrain sites were physically located in different domains of the VTA or SNc. These results suggested that DA cells largely project to one forebrain site and not others. Recently, we used a more sensitive method that enabled brain-wide analysis of the entire axonal arbor of each DA cell subpopulation to show that each cell population in fact does send collaterals to other brain sites, but that the collateralization patterns are largely unique for each subpopulation, and thus the overall projection pattern of each population is largely distinct (Beier et al., 2015, 2019). We also were the first to perform brain-wide input mapping analysis from projection-defined DA populations in the VTA and the adjacent SNc (Beier et al., 2015, 2019; Lerner et al., 2015; Menegas et al., 2015). In contrast to the largely discrete output patterns of these cells, we and others observed that midbrain DA cells receive quantitatively similar inputs from most brain regions, with several biases in the contributions of these inputs onto defined DA cell types. These input biases between conditions may influence the differential role these cells play in subsequent behavioral output, for example in reinforcement behavior (Beier et al., 2015). Given that we have collected whole-brain quantitative datasets of the inputs and outputs of VTA^DA^→NAcMed, VTA^DA^→NAcLat, VTA^DA^→mPFC, and VTA^DA^→Amygdala cells, we wanted to perform a more in-depth analysis to identify factors that differentiated the inputs and outputs of different DA cell types. We previously performed hierarchical clustering on bootstrapped data and demonstrated that VTA^DA^ cells projecting to NAcMed, NAcLat, mPFC, or Amygdala clustered separately based on their output projections to 10 forebrain sites (Beier et al., 2019), indicating that their global output patterns were distinct. We also demonstrated the existence of four groups of output sites with high levels of covariance in our dataset, suggesting that each set of output regions may be preferentially targeted by one DA cell population. However, we did not rigorously identify how these conditions differed and which output sites most contributed to differentiating the projection pattern of each DA cell population.

**FIGURE 1:**
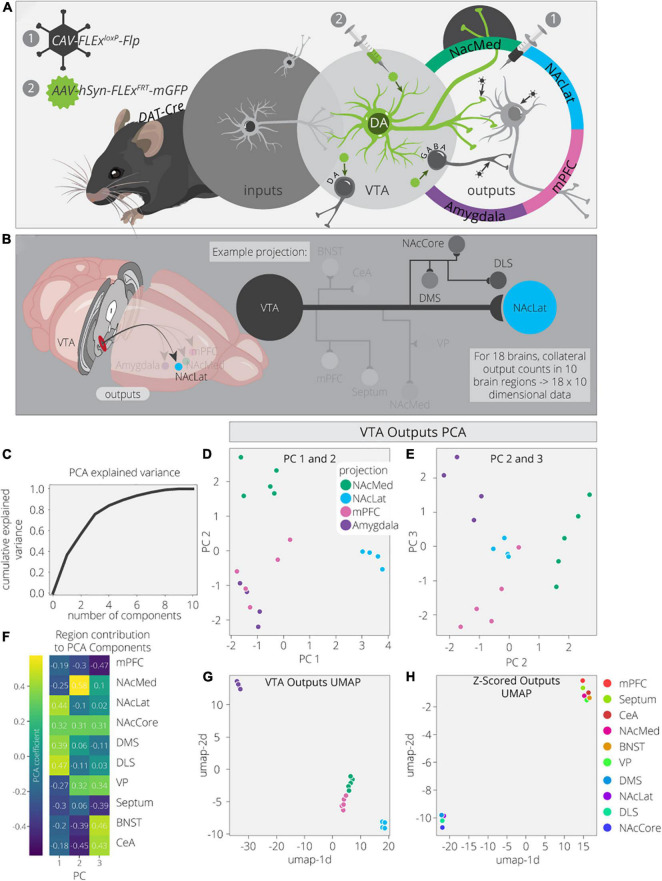
VTA^DA^ outputs are organized by four core projections. **(A)** Schematic for axonal arborization experiments. Viral injections were performed in *DAT-Cre* mice to label collaterals to VTA^DA^ neurons projecting to a specified target. **(B)** Collaterals of VTA projections to the NAcMed, NAcLat, mPFC, and Amygdala were quantified in 10 brain regions across 18 mice. The NAcLat and its major collaterals are highlighted. **(C)** Cumulative explained variance from each principal component. **(D)** Brains are plotted in PCA space for the 1st and 2nd components, colored by projection. **(E)** Brains are plotted in PCA space for the 2nd and 3rd components, colored by projection. **(F)** Heatmap of each output region’s contribution to the first three principal components. **(G)** Brains are plotted in UMAP space, colored by projection. **(H)** Output regions are plotted in UMAP space, embedded with respect to z-scores across mouse brains. Clusters represent outputs with similar patterns of variation across the cohort.

To explore this dataset in greater detail, we first used Principal Component Analysis (PCA) to dimensionally reduce the output data (Figures 1A,B). The output data consist of 18 brain samples from 4 different output-defined conditions (*n* = 5 for NAcMed and mPFC; *n* = 4 for NAcLat and Amygdala). Each sample has 10 measurements, one for each of the output regions quantified. PCA is a linear dimensionality reduction technique that finds a lower dimensional representation of the data that maximizes variance for each principal component (PC). The first PC is a linear combination of the feature space that leads to the highest degree of variance in the data. Each component after makes the same optimization with the remaining dimensions. We found that three components are sufficient to explain ∼70% of the variance in the output data, indicating that these data have a relatively simple structure (Figure 1C). PC1 separates VTA^DA^→NAcLat cells, PC2 separates VTA^DA^→NAcMed cells, PC3 separates VTA^DA^→Amygdala cells, and a combination of PC2 and PC3 separates VTA^DA^→mPFC cells (Figures 1D,E). Thus, three PCs were sufficient to separate each condition.

Next, we wanted to explore how each output region contributed to each PC. For example, PC1, which separated VTA^DA^→NAcLat cells, is driven by NAcLat, nucleus accumbens core (NAcCore), dorsomedial striatum (DMS), and dorsolateral striatum (DLS; Figure 1F). The finding that the NAcLat as an output site helps to differentiate VTA^DA^→NAcLat cells is consistent with the biased projections of each midbrain DA cell population. Additionally, the contribution of other regions in the striatum (except for NAcMed) is consistent with the overall arborization pattern of these cells (Beier et al., 2015). This cell population had the most distinct overall arborization pattern and thus positive weights of these four regions were sufficient to differentiate it. PC2, which separates VTA^DA^→NAcMed cells, is primarily made up of the NAcMed, with smaller contributions from the NAcCore and negative contributions from the mPFC, bed nucleus of the stria terminalis (BNST), and central amygdala (CeA). These negative contributions mean that VTA^DA^→NAcMed cells do not prominently project to the mPFC, BNST, or CeA. Lastly, PC3, which separated VTA^DA^→Amygdala cells, is made up of positive contributions from the ventral pallidum (VP), BNST, and CeA, and negative contributions from the mPFC and septum. The overall arborization patterns of NAcMed-, mPFC-, and Amygdala-projecting VTA^DA^ cells are more similar to one another than to NAcLat-projecting VTA^DA^ cells (Beier et al., 2019); thus in PC2, the negative contributions from the mPFC, CeA, and BNST differentiate NAcMed-projectors from Amygdala- and mPFC-projectors, and in PC3, the negative contributions from the mPFC and septum, which are the brain regions most enhanced in the output targets of VTA^DA^→mPFC cells, differentiate VTA^DA^→mPFC and VTA^DA^→Amygdala cells.

While PCA is useful due to its interpretability, Uniform Manifold Approximation and Projection (UMAP) is better optimized for finding clusters in high dimensional data. Indeed, we find it is much more effective at clustering conditions by output site (Figure 1G; McInnes et al., 2018). As UMAP uses non-linear transformations to achieve clustering, it does not provide us the same detailed information about which output regions are differentiating these clusters. However, we can compute the transpose of the output data and take the z-score to look at how output scores per region vary across samples. Z-scoring normalizes the data such that high and low count regions that have the same variance will have similar values. We used UMAP on these z-scores and found two clusters of output sites with similar variance (Figure 1H). The bottom left cluster contains the four regions that show up in PC1: NAcLat, NAcCore, DMS, and DLS. These data provide confirmation that these four regions vary as a module across these four conditions and serve as a common set of brain sites targeted by the same cell population (VTA^DA^→NAcLat) whereas the other three cell populations share more overlap in their overall projection patterns. This visualization serves as a complement to previous analysis of these data, where hierarchical clustering of the output regions’ covariances found the same organization, highlighting both the robustness of this result and these methods.

To ensure these results were not biased by outputs to the injected projection sites, we removed the projection sites from the output counts and performed the same analysis as before on just the collaterals. We largely see the same clustering behaviors as before (Supplementary Figure 1). The main difference is that the VTA^DA^→NAcMed and VTA^DA^→mPFC brains are harder to separate (Supplementary Figures 1B,C,E). Previously, PC2–now PC3–separated these two conditions the strongest (Figure 1D). This principal component previously had large contributions from three of the projection targets, so it is not surprising that the differences between these conditions are weakened along with the principal component (Figure 1F and Supplementary Figure 1D). Altogether, this analysis confirms that the clustering of projection conditions does not completely depend on including the main projection targets.

### VTA^DA^ Neuron Inputs Do Not Cluster as Cleanly by Projection Site

We and others used intersectional viral-genetic methods to map global inputs to output-defined DA cells (Beier et al., 2015; Lerner et al., 2015; Menegas et al., 2015). While the exact relationships of inputs and outputs varied slightly between different studies, the common finding was that different DA cell populations largely shared common input patterns, with some quantitative differences. We more recently performed a comprehensive mapping of input-output relationships of different cell types in the VTA and reported that (1) the spatial location of cells in the VTA explained a significant amount of variation between conditions for about half of the input sites, (2) cell type did not explain much variation in the inputs between cell populations, and (3) the projection site explained about as much input variation as did spatial position of starter cells in the VTA (Beier et al., 2019). To account for neurons that co-release multiple neurotransmitters, for example glutamate and dopamine, we included the percentage of starter cell immunostaining for tyrosine hydroxylase (TH), a marker of DA neurons, in our linear regression analysis and found it had very little predictive value compared to spatial location (Beier et al., 2019). These observations suggested that the quantitative contribution of inputs a given population of cells receives depends heavily on the physical location of the starter cells in the brain, but not on the identify of what neurotransmitters (e.g., DA, GABA, glutamate) these starter cells release.

**FIGURE 2:**
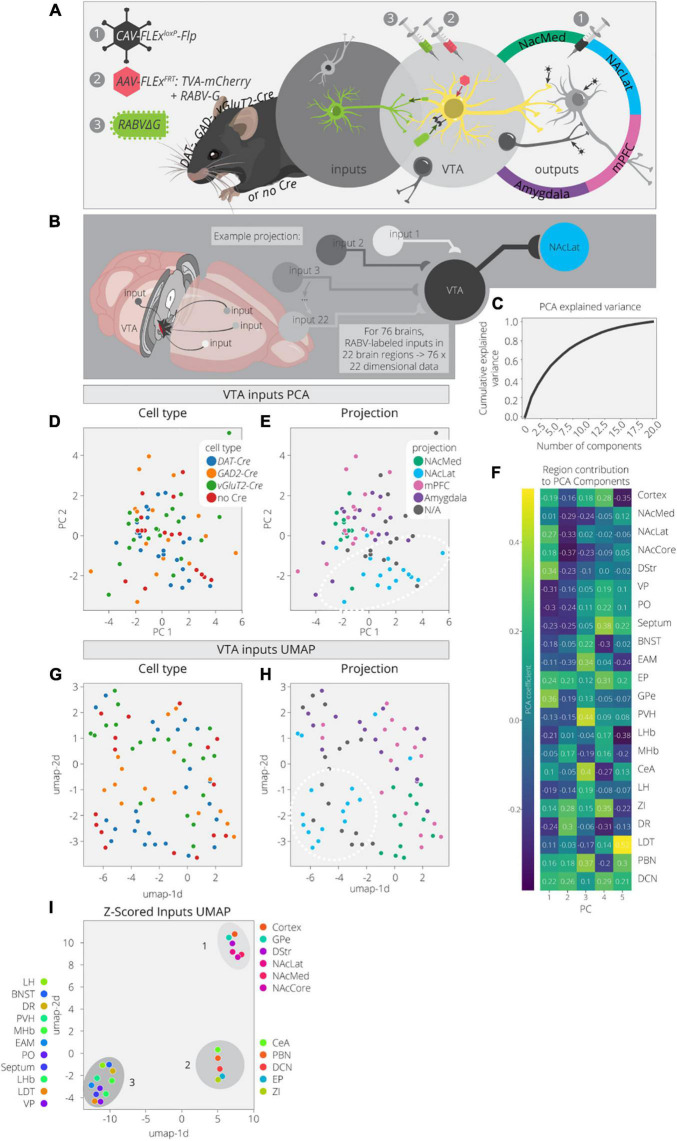
Clusters of VTA inputs revealed by dimensional reduction. **(A)** Schematic for RABV input labeling experiments. *DAT*-, *GAD*-, *vGlut2-Cre*, and non-Cre-expressing mice were used to identify specific (or non-specific) VTA cell types. Injections of *CAV* were used to define output sites. **(B)** Input labeling experiments provided maps of inputs to VTA cells for a combination of different cell-type and projection specifications. Cohort includes 76 brains and 22 input regions counted. **(C)** Cumulative explained variance from each principal component. **(D)** Brains are plotted in PCA space for the 1st and 2nd components, colored by cell type. **(E)** Brains are plotted in PCA space for the 1st and 2nd components, colored by projection. **(F)** Heatmap of each input region’s contribution to the first five principal components. **(G)** Brains are plotted in UMAP space, colored by cell type. **(H)** Brains are plotted in UMAP space, colored by projection. **(I)** Input regions are plotted in UMAP space, embedded with respect to z-scores across mouse brains. Clusters represent inputs with similar patterns of variation across the cohort.

Here we used PCA and UMAP to dimensionally reduce and explore patterns in the input data. These data consist of 76 brains with counts across 22 input regions (Beier et al., 2019). These brains cover a variety of cTRIO and TRIO conditions as well as non-output-defined tracing, resulting in a mix of output and cell-type specifications (Figures 2A,B). A PCA analysis of these data found that three components explained only about 40% of the variance (Figure 2C). This is rather low compared to the output data, even considering the difference in dimensionality, and implies that this dataset is more complex. In the PCA embedding, cell types defined by Cre expression (*DAT-Cre*, *GAD2-Cre*, *vGluT2-Cre*, no Cre) mix together but cells projecting to a common output target do show some organization (Figures 2D,E and Supplementary Figure 2). For example, VTA→NAcLat cells have more positive values in the 1st PC and more negative values in the 2nd PC (Figure 2E). These coordinates reflect higher contributions from brain regions in the basal ganglia which include the NAc, dorsal striatum (DStr), and global pallidus external segment (GPe), as well as lower contributions from the VP and preoptic area (PO) (Figure 2F). Notably, the non-output-defined condition is most similar to the VTA→NAcLat condition, which is expected given that VTA→NAcLat cells comprise the majority of cells in the VTA (Beier et al., 2015).

We next used UMAP to look for any additional clustering behavior between the inputs mapped in different brains in order to assess the similarities and differences between conditions (Figures 2G,H). When defining conditions by Cre expression (*DAT-Cre*, *GAD2-Cre*, *vGluT2-Cre*, no Cre), there are some local neighborhoods within the same conditions, but none are very well-separated into clusters. However, when defining conditions based on output site, the VTA→NAcLat conditions segregate relatively well (Figure 2H). These results are consistent with our previously published analysis (Beier et al., 2019). We then performed a UMAP analysis on the input region z-scores to identify regions with similar variation across conditions (Figure 2I). We found one cluster (cluster 1) made up of inputs from the NAc, DStr, GPe, and cortex. Almost all these regions follow a pattern of contributing positively to the 1st PC and negatively to the 2nd PC (Figure 2F). Thus, these regions provide a stronger fractional innervation to VTA→NAcLat cells than other VTA cells, as observed previously (Beier et al., 2015, 2019). In addition to cluster 1, we observed two other clusters of inputs; one included the CeA, parabrachial nucleus (PBN), zona incerta (ZI), entopeduncular nucleus (EP), and deep cerebellar nuclei (DCN; cluster 2), while the other included all the other regions: VP, PO, LDT, BNST, dorsal raphe (DR), medial habenula (MHb), lateral habenula (LHb), paraventricular nucleus of the hypothalamus (PVH), extended amygdala (EAM), lateral hypothalamus (LH), and septum (cluster 3). These clusters were not readily apparent in our previous analyses of our RABV tracing data, suggesting that there may be additional organization in the input patterns that we had overlooked previously.

### Lateral or Medial Biases of Starter Cells Accounts for Some but Not All VTA^DA^ Input-Output Variation

We previously analyzed the spatial influence of starter neurons in the VTA on the fractional contribution of inputs by using a linear regression test with the medial-lateral and dorsal-ventral coordinates of the starter cell center of mass (COM) (Beier et al., 2019). Since the cells were counted on coronal slices, we do not have nearly as good resolution for the anterior-posterior axis as the ML and DV axes, and for the most part we focus our analyses on these axes. We observed that the medial-lateral coordinate of the COM explained a significant level of variance for about one half of the brain regions across conditions, about the same contribution as the output site and significantly more than the Cre line used to mark starter cells. These results suggested that many inputs to the VTA are biased along the medial/lateral axis in their projections to the VTA, and that the location of the starter cells, as defined by a single point in space, was significantly linked to the fraction of inputs from various brain regions those cells received.

**FIGURE 3:**
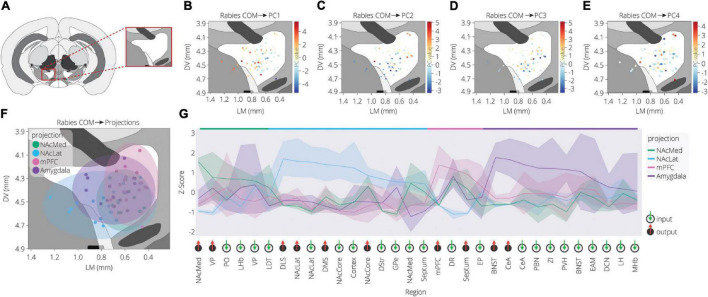
Spatial location of targeted cells in the VTA influences both inputs and outputs. **(A)** Context for coronal slice of VTA used in analysis is shown. **(B)** Brains are plotted by starter cell center of mass (COM), colored by PC1 value. **(C)** Brains are plotted by starter cell COM, colored by PC2 value. **(D)** Brains are plotted by starter cell COM, colored by PC3 value. **(E)** Brains are plotted by starter cell COM, colored by PC4 value. **(F)** Brains are plotted by starter cell COM, colored by projection specification. Ellipsoids are drawn for each condition and have radii of five standard deviations for both dorsoventral and lateromedial axes. **(G)** Z-scores of average input and output counts for each projection condition. Inputs are marked with a green down arrow and outputs with a red up arrow. All inputs and outputs quantified are shown. Inputs and outputs are sorted according to the projection in which they receive the highest z-score. Samples include inputs to and outputs from DA cells only. *p*-values are listed in Table 2.

To further explore the spatial organization of VTA inputs, we plotted each sample according to the starter cell COM and colored them according to their PC values, as calculated in Figure 2 (Figures 3A–E). PC1 has an increasing spatial gradient from the medial to the lateral VTA (Figure 3B). This principal component in general is made up of input populations that project more laterally in the VTA, or to the adjacent SNc/substantia nigra pars reticulata (SNr) (Oh et al., 2014; Beier et al., 2019). This result agrees with the previous finding that the medial-lateral coordinate is related to the fractional contribution from about one half of the input sites examined (Beier et al., 2019). Furthermore, we can compare this spatial organization with the location of VTA→NAcLat starter cells (Figure 3F). The VTA→NAcLat cells are biased toward the lateral side of the VTA, same as the +PC1 cell populations. As PC1 captures the most variation across the data, this means that the primary axis of variation in VTA inputs is whether or not the inputs are biased onto VTA→NAcLat cells, and hence whether the starter cells are located laterally within the VTA or not. PC2 has a mild spatial gradient that increases in the dorsal direction (Figure 3C). PC3, on the other hand, does not have much of a clear spatial bias in the medial-lateral or dorsal-ventral axes (Figure 3D). Rather, starter cell populations with +PC3 span the VTA across the two axes, suggesting that a lack of clear spatial bias in the VTA characterizes this PC. A linear regression analysis confirmed these observations: PC1 was found to have a significant slope in the lateral direction and PC2 in the dorsal direction, while other slopes were not found to be significant after correction for multiple comparisons (Table 1).

**TABLE 1:** Linear regression scores predicting starter cell location from principal components.

PC#	Score	Lateral slope	Significance	Corrected *p*	Dorsal slope	Significance	Corrected *p*
PC1	0.359	8.08	1e-3	1e-2	–2.06	0.175	0.617
PC2	0.299	–2.29	0.031	0.172	–5.36	1e-3	1e-2
PC3	0.0759	0.26	0.81	0.963	–3.08	0.018	0.119
PC4	0.0102	0.04	0.97	0.97	–1.05	0.401	0.871
PC5	0.122	0.4	0.634	0.951	2.9	0.005	0.039

	Corrected *p*-value < 0.05						
	Uncorrected *p*-value < 0.05						

*Slope and p-value for lateral and dorsal coefficients in linear regression models predicting each principal component. p-value is the probability of the coefficient being 0 given the observed data.*

Our analysis with PCA and UMAP separated VTA→NAcLat cells by inputs, but largely failed to differentiate VTA→NAcMed, VTA→mPFC, or VTA→Amygdala cells from one another. To explore the input-output features most specific to each VTA cell type, we stitched together the average input and output counts for each region. We then took the z-score of these values to see how enriched or diminished connections are for that region compared to the other conditions. For each projection condition, we found a unique set of enriched inputs and outputs (Figure 3G and Supplementary Figure 3). Many of these were found significant, even when corrected for multiple comparisons (Table 2). We observed some evidence for reciprocal connectivity: for example, inputs from NAcLat are enriched onto VTA→NAcLat cells, and inputs from the Amygdala and BNST are enriched onto VTA→Amygdala cells that collateralize principally to the BNST, both of which were found to be significant. However, this was not equally clear for all populations, as the NAcMed input was approximately equal onto VTA^DA^→NAcLat and VTA^DA^→NAcMed cells, and we did not observe a preference for cortical inputs onto VTA^DA^→mPFC cells (Beier et al., 2019), suggesting that while some reciprocal connections may exist in the VTA, they may not be universal for all brain regions (Figure 3G).

**TABLE 2:** Sample mean comparison tests for input and output z-scores.

Enriched projection	Region	*p*-value	Corrected *p*-value
NAcMed	NAcMed output	3.31E-09	1.06E-07
NAcMed	VP output	4.93E-02	5.31E-01
NAcMed	PO input	1.50E-01	8.80E-01
NAcMed	LHb input	1.55E-01	8.80E-01
NAcMed	VP input	2.25E-01	8.99E-01
NAcMed	LDT input	6.07E-01	9.74E-01
NAcLat	DLS output	7.43E-07	2.23E-05
NAcLat	NAcLat output	2.57E-05	6.94E-04
NAcLat	NAcLat input	3.00E-04	7.76E-03
NAcLat	DMS output	4.69E-04	1.17E-02
NAcLat	NAcCore input	2.47E-03	5.54E-02
NAcLat	Cortex input	6.15E-03	1.27E-01
NAcLat	NAcCore output	6.09E-02	5.85E-01
NAcLat	DStr input	1.57E-01	8.80E-01
NAcLat	GPe input	2.69E-01	9.19E-01
NAcLat	NAcMed input	2.78E-01	9.19E-01
NAcLat	Septum input	3.64E-01	9.34E-01
mPFC	mPFC output	8.87E-06	2.48E-04
mPFC	DR input	9.29E-03	1.62E-01
mPFC	Septum output	1.51E-02	2.28E-01
mPFC	EP input	5.19E-01	9.74E-01
Amygdala	BNST output	3.35E-08	1.04E-06
Amygdala	CeA output	2.93E-06	8.50E-05
Amygdala	CeA input	1.23E-03	2.91E-02
Amygdala	PBN input	6.24E-03	1.27E-01
Amygdala	ZI input	7.06E-03	1.32E-01
Amygdala	PVH input	1.14E-02	1.86E-01
Amygdala	BNST input	2.15E-02	2.93E-01
Amygdala	EAM input	1.97E-01	8.89E-01
Amygdala	DCN input	5.69E-01	9.74E-01
Amygdala	LH input	7.01E-01	9.74E-01
Amygdala	MHb input	8.77E-01	9.74E-01

	Corrected *p*-value < 0.05		
	Uncorrected *p*-value < 0.05		

*Significance tests comparing projections for each input and output. For each input and output, the sample mean of the most enriched projection was compared against the remaining projections with a T-test. p-values are corrected for multiple comparisons using a Bonferroni correction.*

The input and output sites enriched onto VTA^DA^→NAcLat cells consist of those previously identified (Beier et al., 2015, 2019) and shown in Figures 1, 2. However, we also found a number of brain sites enriched as inputs to or outputs from VTA^DA^→Amygdala cells that we did not previously identify. These outputs include preferential projections to the Amygdala and BNST, as previously described (Beier et al., 2019), as well as inputs from the CeA, PBN, ZI, PVH, BNST, EAM, DCN, LH, and MHb. Many of these brain regions, including the CeA, PBN, PVH, BNST, and EAM, are in the extended amygdala and are principally involved in stress and anxiety-related behaviors (Bernard and Besson, 1988; Han et al., 2015; Chou et al., 2018; Zhou et al., 2018; Chiang et al., 2019). These same regions are also the strongest positive contributors to PC3 (Figure 2F). Furthermore, the location of starter cell COM with a +PC3 (Figure 3D) most closely mirrored the distribution of VTA→Amygdala cells, which are distributed broadly throughout the VTA with a centroid in approximately the middle of the structure (Figure 3F). These visualizations therefore provide further evidence of the spatial organization of inputs on the VTA that we reported previously, and they also suggest the existence of subpopulations of VTA cells that receive preferential inputs from key brain regions involved in the brain’s stress response.

To explore how starter cell COM and RABV input cells distinguish the various projection conditions, we trained logistic regression models to predict each condition. Logistic regression can be used for multiclass classification, in which a logistic regression model is trained for each condition, and the condition with the highest probability is assigned to a given observation. We used the first five principal components as features representing the inputs to the VTA, to reduce overfitting our dataset and to simplify the model to increase the model’s interpretability. We trained models on the principal components and the starter cell COMs separately, and on both combined. Unsurprisingly, projection conditions already grouped together in the PCA plots were well-predicted by the principal components, for example the VTA→NAcLat and VTA→Amygdala cell populations (Table 3). Additionally, projection conditions that appeared to have a spatial bias achieved higher scores when predicted by COMs, for example the VTA→NAcLat and VTA→mPFC. VTA→NAcMed was predicted greater than chance across each individual set of features. It also ends up with one of the highest prediction scores when both PCs and COMs are considered. This result suggests that a combination of input features and spatial location is needed to encode the identity of this population. Logistic regression models are highly interpretable, as each feature is assigned a coefficient which models the increased or decreased likelihood of a label given a higher or lower value of the feature. These coefficients largely recapitulate observations we have already made. For example, PC1 is useful for predicting VTA→NAcLat, PC3 is useful for predicting VTA→Amygdala (Supplementary Figure 4A), and the medial-lateral coordinate is useful for predicting VTA→NAcLat and VTA→mPFC populations (Supplementary Figure 4B). In the model incorporating both PCs and COMs, we found that a combination of PC1 with the dorsal and anterior coordinates can predict the VTA→NAcMed condition (Supplementary Figure 4C). These analyses imply that while the most striking aspect of VTA input connectivity is the presence of spatial gradients, there may be some interesting connectivity relationships that are not uniquely delineated by a medial-lateral or dorsal-ventral gradient.

**TABLE 3:** Logistic regression scores predicting projection conditions from starter cell location and principal components.

Projection	3 PCs	5 PCs	COMs	5 PCs + COMs
NAcLat	0.8125	0.875	0.6875	0.8125
NAcMed	0.5	0.333333	0.5	0.75
mPFC	0.375	0.4375	0.625	0.625
Amygdala	0.625	0.625	0.4375	0.75
None	0.5625	0.4375	0.125	0.6875

	Score > 0.8			
	Score > 6			
	Score > 0.4			

*Logistic regression model scores predicting each condition using RABV input principal components and/or starter cell location, using multi-class classification.*

### Spatial Analysis of Allen Mouse Connectivity Atlas Data Finds Archetypal Projection Patterns to the Ventral Tegmental Area

Using publicly available data from the Allen Mouse Brain Connectivity Atlas, we had previously investigated the spatial organization of projections to the VTA. We had found that the relative projection ratio across some inputs varied across the lateral-medial axis and that was related to the relative ratio of inputs received by different VTA^DA^ cell populations, linking the density of projections from a given input site to RABV-labeled inputs (Beier et al., 2019). However, this analysis was done with a limited set of brain regions, focused only on the medial-lateral gradient along the VTA, and only explored the link between input density and DA neurons in the VTA. Here we wanted to explore this question with a broader perspective and assess the relationship between projections throughout the ventral midbrain from each of the input sites that we quantified in our previous studies. We wanted to assess globally how closely spatial projection patterns throughout the ventral midbrain relate to RABV input mapping datasets.

**FIGURE 4:**
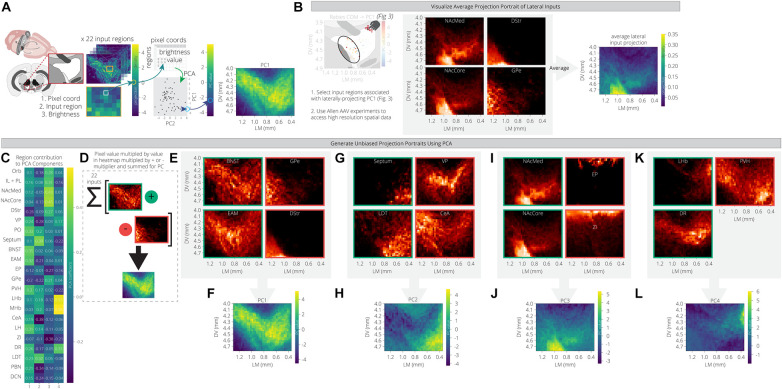
PCA of data from the Allen Mouse Brain Connectivity Atlas reveals projection archetypes across different input groups. **(A)** Schematic of analysis. Sample projections to the VTA were pulled from the Allen Mouse Connectivity Atlas for 22 input regions. Pixel values representing projection density were pulled from the coronal slice for each region to generate a table of 2,058 pixel coordinates x 22 regions. PCA found a linear combination of input regions that maximizes variation across the pixels. Each pixel is then visualized on a coronal slice of the VTA with its PC value, revealing the most common projection portrait. **(B)** Brain regions that preferentially provide inputs to VTA→NAcLat cells were selected as a test case to see if they might have a common projection pattern. Sample projections from these regions to the VTA are shown from the Allen data, along with their average projection portrait. **(C)** Heatmap of each input region’s contribution to the first four principal components. **(D)** Positive and negative contributing regions to each principal component are summed according to their sign in order to generate the principal component projection portrait. **(E–L)** Example projection portraits are shown for the major positive and negative contributing regions for each of the first four principal components. The projection archetypes corresponding with these principal components are shown in panels **(F,H,J,L)**.

For each input region, we selected three experiments from the Allen Mouse Brain Connectivity Atlas and took the average projection into the ventral midbrain. The NAcLat was excluded as an input site, as the Atlas does not contain injections into this site. We also used injections in the infralimbic/prelimbic (IL/PL) and orbitofrontal cortex (Orb) to represent two distinct regions of the anterior cortex. We then mapped these projections onto a coronal slice of the ventral midbrain to facilitate visualization. We used an extended spatial domain that allowed us to assess projections within the VTA as well as to adjacent structures. As before, we used PCA to reduce the dimensions of this space. The first principal component is a weighted combination of the projections from the 22 input sites that maximizes variance across the ventral midbrain window. This weighted combination can then be visualized in the original spatial dimensions. By comparing the PC projection patterns with the region contributions to the PCs (Figure 4A), we can see what the archetypal projection patterns are and how input region projections are similar or dissimilar. For example, we computed and plotted the archetypal projection of four regions that provide preferential inputs onto VTA→NAcLat cells: The NAcMed, NAcCore, DStr, and GPe. This archetype shows a projection to the lateral VTA, where the VTA→NAcLat cells are located, as expected (Figure 4B).

PC1 includes projections that relatively uniformly innervate the entire VTA, with little bias (Figures 4C–E). This marks the +PC1 pixels, and thus we would expect the regions with positive contributions to this PC to have higher projections over this space. Some example input sites with this pattern include the PO, BNST, EAM, PVH, and LH (Figure 4C). Interestingly, these regions all fall within cluster 3 of our RABV data (Figure 2I) and have inputs that are enriched onto VTA→Amygdala cells (Figure 3G). Another characteristic of PC1 is that its negative values are ventral and lateral to the VTA. We therefore expect -PC1 pixels to have higher projections from the -PC1 regions and lower projections from the +PC1 regions. The DStr and GPe both do not project much to the VTA directly, but they do have strong projections lateral to the VTA (Figure 4E). Likewise, the +PC1 regions – PO, BNST, EAM, PVH, and LH – tend not to project at all to this area lateral and ventral to the VTA, but rather project broadly throughout the VTA. Thus, the primary axis of variation that PC1 seems to capture contains the regions that are projecting with little bias to the VTA, and those that are projecting lateral and ventral to the VTA (Figures 4D,F). This projection primarily innervates VTA→Amygdala cells that are distributed throughout the VTA (Figure 3F).

+PC2 receives the strongest weights from the septum, LDT, PO, MHb, and LHb, while -PC2 is composed primarily of the VP, GPe, CeA, PBN, and DCN (Figure 4C). This PC appears to have a medial/ventral bias, as the brain regions with the strongest weights project primarily to the medial and ventral portion of the VTA, while the GPe, CeA, PBN, and DCN all project laterally/dorsally (Figures 4G–H). Given that the VTA→NAcMed cells are located the furthest in the ventromedial portion of the VTA (Figure 3F), we would expect that VTA^DA^→NAcMed cells receive preferential input from these brain regions. Indeed, the PO, LHb, and LDT preferentially connect to VTA^DA^→NAcMed cells, while the septum connects approximately equally to VTA→NAcMed and VTA→NAcLat cells (Figure 3G). Notably, -PC2 receives a relatively strong negative weight from the DR (Figure 4C).

+PC3 is primarily composed of inputs from the basal ganglia (NAcMed, NAcCore, DStr, GPe), and the two cortical regions, IL/PL and Orb, while –PC3 is composed primarily of the EP, ZI, and DCN (Figure 4C). +PC3 corresponds to inputs that project ventrolateral to the VTA (Figures 4I,J), and primarily innervate VTA→NAcLat cells (Figures 2H, 3G). The brain regions that contribute to -PC3 project dorsal to the VTA.

+PC4 has strong contributions from the LHb, MHb, and DR, while -PC4 is primarily composed of the septum, PVH, and ZI (Figure 4C). Of the positive contributors, the LHb and MHb are also present in +PC2 as they broadly project to the medial VTA, which also is where VTA^DA^→NAcMed cells are located. In contrast, the DR contributes mostly to +PC4 and +PC1. This combination of PCs describes the DR’s broad projection to the dorsal VTA with a strong bias to the dorsomedial VTA. +PC4’s archetypal projection is also to the dorsomedial VTA (Figures 4K,L), a region that most prominently includes VTA^DA^→mPFC neurons (Figure 3F). Accordingly, the DR preferentially innervates VTA^DA^→mPFC cells (Figure 3G) and thus is the main input brain region that differentiates VTA^DA^→NAcMed from VTA^DA^→mPFC cells.

These data demonstrate that the first four PCs using data from the Allen Mouse Brain Connectivity Atlas correspond to the input biases of the four VTA populations that we examined here. Therefore, our conclusion is that we can recapitulate the principal differences in inputs to different cell populations in the VTA solely by identifying the archetypal projections into the VTA using open-source data from the Allen Institute.

### Patterns of Input Innervation Are Conserved Between RABV Mapping and Allen Projection Data

**FIGURE 5:**
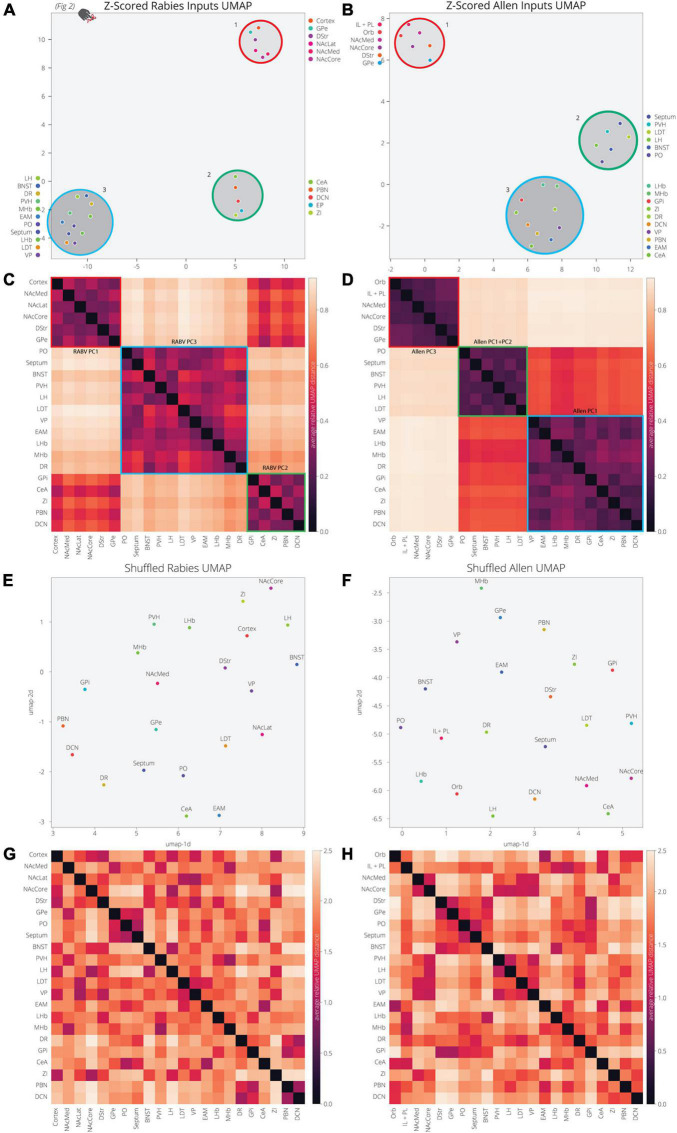
UMAP dimensional reduction of RABV and Allen data reveal common clusters of VTA inputs. **(A)** Input regions are plotted in UMAP space, embedded with respect to z-scores from the RABV input mapping data. Clusters represent inputs with similar patterns of variation across the cohort. **(B)** Input regions are plotted in UMAP space, embedded with respect to z-scores across pixels in the Allen data. **(C)** Heatmap of pairwise distances (averaged across 20 UMAP embeddings) for the RABV input data. Regions are grouped according to hierarchical clusters. Clusters are highlighted to match the clusters above in the UMAP plot; they are also annotated according to which principal components to which the regions contribute. Regions are grouped to line up with the Allen clusters. **(D)** Heatmap of pairwise distances (averaged across 20 UMAP embeddings) for the Allen input data. **(E–H)** Same plots as panels **(A–D)**, but UMAP was run on scrambled data. For each region, z-score values were scrambled across mouse brains for RABV data, or pixel coordinate for the Allen data.

Our analysis of the Allen’s projection data suggests that we can recapitulate the variance in RABV mapping experiments by decomposing the Allen’s projection data into principal components. As we previously mentioned, UMAP is better optimized for identifying the relationship between variables in high-dimensional space. We therefore wanted to assess the relationship between input sites to the VTA, defined either through their covariance in our RABV mapping data or spatial similarity in Allen projection data. We demonstrated earlier that the input sites in RABV mapping experiments segregate into three clusters (Figures 2H, 5A). As UMAP embeddings can be somewhat stochastic because they rely on initial seeding conditions, we computed the distance between points relative to the maximum distance between any two points in each embedding, over 20 embeddings, then averaged across all embeddings (Figures 5A–D). In both cases, we identified three clusters of brain regions. Cluster 1 contained perfect correspondence between RABV and Allen datasets, and included regions in the frontal cortex (either anterior cortex or both the IL/PL and Orb), NAcMed, NAcCore, DStr, and GPe (the NAcLat was not included in the Allen dataset). While clusters 2 and 3 in the RABV and Allen datasets did not perfectly align, they did have similar structures. RABV cluster 2 included the CeA, EP, ZI, PBN, and DCN. These regions also clustered together in the Allen data, but were joined by the VP, EAM, LHb, MHb, and DR that split from cluster 3. The remainder of the brain regions (septum, BNST, PO, PVH, LH, LDT) are in cluster 3 for both datasets. Notably, the distance between clusters 2 and 3 in the Allen data is much smaller than to cluster 1 and thus, Allen clusters 2 and 3 have a more similar projection profile to each other than to cluster 1. Overall, we observed substantial similarity between RABV and Allen datasets, suggesting that the covariance in input labeling using RABV mapping can be largely attributed to differences in axonal innervation from input sites and thus, the information can be gleaned through parsing open-source projection datasets. To demonstrate that these associations were not attributed to chance, we scrambled the association of the COM with fraction of inputs labeled in the RABV dataset, or the order of z-scores for pixel intensity for each input site. UMAP was unable to identify clusters or significant levels of co-variance in either scrambled dataset (Figures 5E–H), demonstrating that the high covariance between selected input brain sites is highly significant and similar between both RABV and Allen datasets.

## Discussion

**FIGURE 6:**
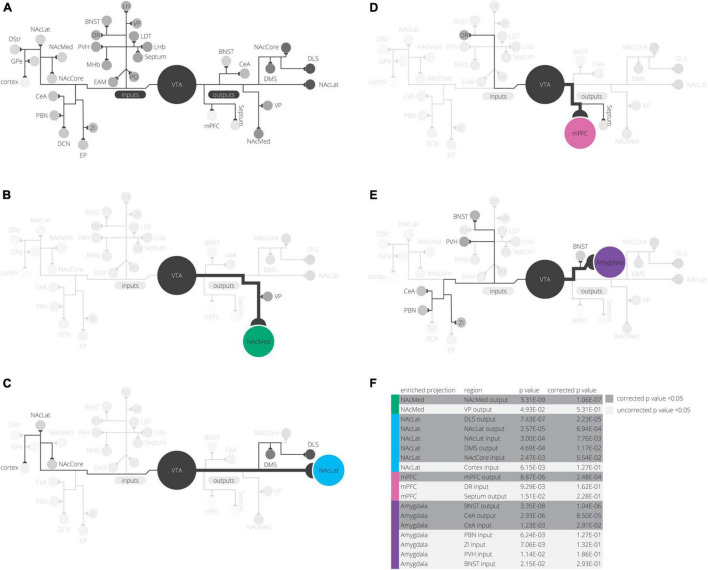
Summary of findings. **(A)** All inputs and outputs we mapped in our experiments are shown, grouped in their clusters from UMAP analysis. **(B–E)** For each projection, elevated inputs and outputs are shown (according to Figure 3G). **(F)** Inputs or outputs significantly elevated in any projection condition are shown grouped by projection. *p*-values are corrected for multiple comparisons using a Bonferroni correction.

Our detailed observations of input and output datasets of VTA cells revealed several interesting findings. The largest contributor to variance in our input tracing dataset is the medial-lateral gradient in the VTA, which differentiates the VTA^DA^→NAcLat cells from the other three subpopulations. The VTA^DA^→NAcLat cells also had the most distinct collateralization pattern of the four VTA^DA^ subpopulations studied. These results confirm our previous analyses (Beier et al., 2015, 2019). However, here we were able to further differentiate the VTA^DA^ projections to the NAcMed, mPFC, and Amygdala by inputs as well as outputs with an integrated spatial analysis of several high dimensional datasets. By exploring the z-scores of input counts in different brain regions, we found that the PO, LHb, VP, and LDT inputs were elevated for VTA^DA^→NAcMed cells, DR and EP inputs are elevated for VTA^DA^→mPFC cells, and CeA, PBN, ZI, PVH, BNST, EAM, DCN, LH, and MHb inputs are elevated for VTA^DA^→Amygdala cells (Figure 6). The z-score normalization allowed us to find elevations in inputs and outputs whose fractional counts were smaller in magnitude than other regions. Logistic regression models demonstrated how RABV inputs and starter cell location contributed to differentiating these conditions. Investigation of the projection patterns of inputs to the VTA revealed that VTA input populations can be differentiated into several projection archetypes—projections to the VTA broadly, projections to regions around but not including the VTA, and projections to subdomains of the VTA. Lastly, we showed that the patterns of these projection archetypes mirror input differences to VTA subpopulations. These data together demonstrate that the location of different DA cell populations determines the quantitative contribution from different inputs and, thus, the signals that these cells receive.

### Comprehensive Quantitative Analysis Enables Differentiation of Four VTA^DA^ Cell Populations

Our goal in this study was to identify input and output factors that differentiate VTA^DA^ neurons. Previous studies have shown that subpopulations of VTA^DA^ cells differ in their forebrain projections, electrophysiological properties, and behavioral functions (Lammel et al., 2008, 2011; Kim et al., 2016). Comprehensive input-output mapping studies from us and several other groups suggested that DA cell populations received inputs from the same brain regions in quantitatively similar proportions, with some biases. Of note, we previously found that VTA^DA^ cells projecting to the NAcLat received more inputs from the striatum and globus pallidus external segment than the other VTA^DA^ cells that we examined (Beier et al., 2015, 2019). This is likely because the VTA^DA^→NAcLat cells are located the most laterally within the VTA, and most of the basal ganglia inputs project most strongly to the adjacent SNr (Beier et al., 2015, 2019). While our previous analyses comparing the fractional contribution from 22 input sites to 4 different VTA^DA^ cell populations were able to differentiate VTA^DA^→NAcLat cells, VTA^DA^ cells projecting to the NAcMed, mPFC, or Amygdala appeared highly similar. Here, by exploring the z-scored input and output data, we identified sets of inputs and outputs elevated for each cell type.

First, we observed that some VTA^DA^ cell types may be preferentially reciprocally connected, including the predominant VTA^DA^→NAcLat subpopulation. While this was not the case for all our observed cell populations, as VTA^DA^→mPFC cells received fewer mPFC inputs than did VTA^DA^→NAcLat cells (Beier et al., 2019), it does suggest that the hypothesis of reciprocal connectivity cannot entirely be discarded. A model of reciprocal connectivity was proposed long ago (Swanson, 1982; Alheid and Heimer, 1988; Zahm, 2006; Yetnikoff et al., 2014), but a recent viral-genetic mapping study failed to find evidence for this reciprocal connectivity in the VTA (Menegas et al., 2015). By comparing the average percent of inputs arising from individual identified brain regions across animals, we also failed to observe statistically significant evidence of reciprocity (Beier et al., 2015, 2019). However, our z-scored analysis gave better visualizations of the lower fractional inputs, supporting the possibility that some preferential reciprocal connectivity may exist in the VTA. This observation argues that a detailed and higher powered investigation into reciprocal connections in RABV mapping datasets may be necessary to reveal the true connectivity relationships in the brain. It is also possible that reciprocal connections may be more present in certain structures and projections than others. It is however noteworthy that in order for these analyses to achieve significance, comparatively larger datasets like ours may be needed, whereas the majority of RABV mapping studies use only a handful of animals (typically 6 or fewer) per condition.

Second, input regions that are integrated into common circuits and have been implicated in common behavioral functions tend to provide preferential innervation onto one particular VTA^DA^ cell type. For example, striatal and globus pallidus inputs that comprise key components of the basal ganglia preferentially provide input to VTA^DA^→NAcLat cells that project back into the striatum. We also found that several regions in the extended amygdala that have been implicated in stress-related behaviors preferentially provide input onto VTA^DA^→Amygdala cells. Several studies have been published in the past few years about the role of VTA^DA^→Amygdala cells in reward and aversion learning, fear learning, as well as anxiety (Lutas et al., 2019; Lin et al., 2020; Tang et al., 2020). The CeA, PBN, ZI, PVH, BNST, and EAM all play key roles in aversion and anxiety behaviors (Bernard and Besson, 1988; Han et al., 2015; Chou et al., 2018; Zhou et al., 2018; Chiang et al., 2019), and interestingly, all contain neurons that express CRF, a neuropeptide that modulates DA cells in the midbrain and DA responses in downstream structures (Ungless et al., 2003; Wanat et al., 2008; Lemos et al., 2012). While each of these brain regions participates in behaviors other than fear learning and anxiety, it is interesting that each of these regions, which are distributed throughout the brain, has a similar projection pattern in the VTA. This suggests that these regions may work in concert to facilitate behavioral outcomes associated with stress and aversion/fear learning through VTA^DA^→Amygdala cells. The preferential inputs from basal ganglia regions to VTA^DA^→NAcLat cells and stress-related inputs to VTA^DA^→Amygdala cells is likely due to the fact that inputs with common functions form particular projection archetypes. This means that inputs with a similar function may share a set of factors that govern their connectivity, an idea that we explore further below.

Third, variance in our input and output data can be explained by differences in the location of starter cells within the VTA. The input regions that provided preferential innervation to particular VTA^DA^ cell populations preferentially innervated regions of the VTA that matched the spatial location or distribution of the corresponding VTA^DA^ cell type. These results reinforce our previous conclusion that organization within the VTA is largely spatial, with cell type providing little influence on the inputs that those cells receive (Beier et al., 2019). However, they also highlight that additional dimensions of spatial pattern exist within the VTA beyond the medial-lateral gradient that we identified earlier and that these patterns underlie differences in inputs that each cell population receives. For example, we found that while VTA^DA^→NAcMed and VTA^DA^→mPFC cells were both located medially in the VTA, inputs that were ventrally biased in the medial VTA preferred VTA^DA^→NAcMed cells, and inputs that were dorsally biased preferred VTA^DA^→mPFC cells. These results indicate that these spatial preferences matched the relative ventral or dorsal bias of these VTA^DA^ subpopulations, respectively (Figure 3). We also found that VTA^DA^→Amygdala cells had the broadest medial-lateral distribution and were located the most centrally in the VTA. Inputs to these cells also lacked clear medial-lateral biases. Altogether, our conclusions in this study are entirely consistent with our previous conclusions, while also extending them by identifying more subtle differences in the location of DA cells within the VTA as well as the location of input projections throughout the VTA.

### Specificity of RABV Transmission and Implications for Rules Governing Connectivity

As we noted above, comparing the averages between the percentage of inputs received from different brain regions across animals was sufficient only to reveal the largest differences between conditions. In our dataset, this was sufficient to differentiate VTA^DA^→NAcLat cells from the rest, but insufficient to parse apart VTA^DA^→NAcMed, VTA^DA^→mPFC, and VTA^DA^→Amygdala cells from one another. Notably, the method of comparing averages across animals is the standard method of analysis of RABV mapping datasets. Beyond being the simplest approach to analyzing these data, most mapping datasets likely contain too few samples to effectively perform PCA or UMAP analyses of their data. This is likely because RABV mapping experiments are labor intensive and typically not performed on the scale that ours was. In the case of our 76-brain dataset, it took years of viral generation, mouse breeding, stereotaxic injection, brain sectioning, imaging, and manual quantification to obtain it. That it is currently a one-of-a-kind dataset has provided a unique opportunity to explore connectivity within the VTA as well as assess the merit of different analyses of RABV mapping datasets.

It is also worth assessing what RABV mapping studies can tell us and what they cannot. The prevailing viewpoint among those who use RABV circuit mapping is that RABV transmits between neurons in a synapse-specific fashion. We have argued that the evidence for synaptic-exclusive transmission of RABV is weak (Beier, 2019, 2021; Rogers and Beier, 2021). The fact that the results from RABV mapping experiments such as we conducted in the VTA can be largely recapitulated only from anterograde mapping experiments such as those from the Allen Brain Institute, notably ones that do not differentiate axons of passage from axons that functionally innervate cells in the VTA, could be an additional argument that RABV can spread non-specifically. However, we previously performed an experiment in the VTA that showed that RABV transmission from one cell to another is quite different from direct injection of RABV (Beier et al., 2019). This result was also seen in a similar set of experiments carried out in the DMS (Wall et al., 2013). That a quantitatively different set of inputs was obtained from tracing experiments utilizing different modes of RABV administration provides a strong argument that one-step RABV mapping is not equivalent to directly administering RABV into the brain.

Our observation thus is that RABV mapping does not reveal cell type-specific connectivity, as defined by spatially intermingled cells defined by neurochemical identity. In assessing the implications of this finding, it is worthwhile to consider our state of knowledge regarding spatial patterning and mechanisms that govern connectivity between neurons in the brain. Spatial patterning within the brain during development has been extensively studied, and the roles of families of patterning molecules such as ephrins, netrins, slits, and semaphorins have been well documented (Yu and Bargmann, 2001; Bashaw and Klein, 2010). Other surface proteins such as Teneurins, Tolls, DIPs, and Dprs may play roles in regulating connectivity at the cellular level (Hong et al., 2012; Ward et al., 2015; Barish et al., 2018). However, our understanding of the exact roles that these surface proteins play in dictating whether or not two neurons form connections, particularly in the rodent brain, is limited. It is important to note that we do not know the biases that RABV may have for spread to particular cell types in the brain, and it is possible that these biases are similar for all cell types and outweigh any actual differences in connectivity. Advances in RABV mapping technology, for example the development of a genetically barcoded RABV, may enable the exploration of the role that classes of surface proteins may play in defining connections between neurons (Saunders et al., 2021). However, it is also possible that the lack of cell type-specific connectivity revealed by RABV may be biologically meaningful. Such random connectivity patterns would then have implications for how connections at both the macro and micro-scales influence circuit output and animal behavior.

## Future Directions

We and others have extensively mapped inputs and outputs of cells in the ventral midbrain and have detailed the role of spatial location in determining input patterns between different cell types (Beier et al., 2015, 2019; Lerner et al., 2015; Menegas et al., 2015). Our analysis in this study extends our previous observations. One next step is to determine if this finding applies to brain regions outside of the VTA. The observation that spatially intermingled cell populations tend to receive inputs from the same brain regions in quantitatively similar proportions supports the hypothesis that spatial location is the major determinant of global input patterns, at least as measured by one-step RABV mapping. However, the sources of spatial patterning of inputs and projection archetypes remain unknown. That brain regions sharing a common behavioral role have a similar projection pattern throughout the ventral midbrain suggests that these regions likely follow similar rules of patterning in the ventral midbrain, and this patterning in turn guides their preferential connectivity into particular cell types within the ventral midbrain. The identification of patterning molecules expressed during development and synapse formation through single cell RNA sequencing, for example, would help to elucidate what molecular pathways dictate projection patterns. It would also be interesting to test how ubiquitous this phenomenon of projection archetypes is throughout the brain and if it relates to projection-defined cells in a similar way as in the VTA. If so, the definition of projection archetypes during development along with spatial localization of projection-defined cell types may be one important generator of specificity in circuit connectivity in the brain.

## Materials and Methods

### RABV Input and Axonal Arborization Output Tracing

Input and output mapping from VTA cells was described previously (Beier et al., 2015, 2019). Briefly, *DAT-Cre*, *GAD2-Cre*, *vGluT2-Cre*, and wild type C57Bl/6 mice were obtained and housed with 12 hour light/dark cycles and food and water *ad libitum* (Beier et al., 2019). Viral vectors were prepared as previously described (Schwarz et al., 2015). For TRIO experiments, *CAV-Cre* was injected into an output site, and Cre-dependent AAVs expressing the avian TVA protein as well as the rabies glycoprotein, RABV-G, were injected into the VTA. Two weeks later, EnvA-pseudotyped rabies virus (RABV) was injected into the VTA. These TRIO experiments thus labeled the inputs to VTA neurons with a specified output. We also performed cell-type specific TRIO (cTRIO) experiments. This included injecting a *CAV-FLEx*^loxP^*-Flp* into a target output site and Flp-dependent AAVs expressing TVA and RABV-G into the VTA, and EnvA-pseudotyped RABV 2 weeks later. These cTRIO experiments labeled inputs to VTA neurons of a specific cell-type with a specified output. Rabies labeling experiments were also performed to cover conditions without an output target specified.

Axonal arborization experiments labeled the axons of VTA neurons with projections to a specified target. We performed similar CAV and AAV injections to the above, but rather than TVA and RABV-G we expressed a membrane-targeted GFP in targeted cells. This allowed us to view the entire axonal arbor of these cells. After 2 months, animals were perfused with PBS and 4% formaldehyde. For inputs, cells were counted manually using preselected regions. For both inputs and outputs, data were normalized by the total counts in each brain, accounting for differing levels of viral infection. Detailed protocols for input tracing and axon arborization can be found in previous publications (Beier et al., 2015, 2019).

### Region Selection

Regions were selected for RABV input and axonal arborization output tracing according to previous publications (Beier et al., 2015, 2019). Notably, for VTA inputs we subdivided the global pallidus into the global pallidus external (GPe) and the entopeduncular nucleus (EP), the rodent equivalent of the GPi. For outputs, we subdivided the dorsal striatum into the dorsal lateral striatum (DLS) and dorsal medial striatum (DMS). Since the DLS does not substantially project to the VTA, and since the divide between the DMS and DLS is somewhat arbitrary, we did not subdivide the DStr for inputs. Here and previously we binned the anterior cortex into a single region. We previously subdivided the cortex into its composite regions, but did not find biased projections onto VTA cells according to cell type or projection (Beier et al., 2019). We did explore some substructures in the Allen Mouse Brain Connectivity Atlas analysis, including the orbital cortex, and the combined infralimbic and prelimbic cortical regions. For the amygdalar regions, we analyzed the central amygdala as an input site. For the projection site, we targeted the CeA, but we were not confident that our injections were completely restricted to this site, and hence we call these amygdala-projecting cells. It is likely that our VTA injections did not substantially induce DA cells located in the retrorubal field (RRF), where some have detected projections to amygdalar structures (Zahm et al., 2011).

Groupings of brain regions are listed below, in alphabetical order:

CeA–central amygdala lateral, medial, and capsular nuclei
Cortex–anterior cingulate cortex (ACC); infralimbic cortex (IL); insular cortex (Ins); motor cortex (MO; anterior portion); orbital cortex (Orb); prelimbic cortex (PL); somatosensory cortex (SS, anterior portion). This is the same composite structure as called the anterior cortex in Beier et al. (2015, 2019).
DR–as defined in Weissbourd et al. (2014).
EAM–anterior amygdaloid area, basomedial amygdala, anterior cortical amygdaloid nucleus, cortex-amygdala transition zone
LDT–laterodorsal tegmental area, dorsomedial tegmental area, dorsal tegmental nucleus, Barrington’s nucleus, ventral tegmental nucleus, subpeduncular tegmental nucleus
PO–medial preoptic area, lateral preoptic area, lateral anterior hypothalamic area, anterior hypothalamic area, striohypothalamic nucleus
Septum–triangular septal nucleus, lateral septum, dorsal fornix, septofimbrial nucleus, medial septum, septohypothalamic nucleus, septohippocampal nucleus, lambdoid septal zone
VP–interstitial nucleus of posterior limb of anterior commissure (IPAC), substantia innominata, horizontal diagonal band, nucleus of the vertical diagonal band

Abbreviations for brain regions made throughout the paper are listed below, in alphabetical order:

BNST–bed nucleus of the stria terminalis
CeA–central amygdala
DCN–deep cerebellar nucleus
DR–dorsal raphe
DStr–dorsal striatum
EAM–extended amygdala
EP–entopeduncular nucleus (GPi)
GPe–globus pallidus (GPe)
LDT–laterodorsal tegmentum
LH–lateral hypothalamus
LHb–lateral habenula
MHb–medial habenula
NAcCore- nucleus accumbens, core
NAcMed–nucleus accumbens, medial shell
NAcLat–nucleus accumbens, lateral shell
PBN–parabrachial nucleus
PO–pre-optic area
PVH–paraventricular hypothalamus
VP–ventral pallidum
VTA–ventral tegmental area
ZI–zona incerta

### Dimensional Reduction of Output and RABV Input Data

Principal Component Analysis (PCA) was used to dimensionally reduce both axon arborization output and RABV input data. PCA is a linear dimensional reduction technique that finds the maximal axes of variation through a dataset. Once a PCA embedding is found, each principal component can be unpacked to find out what linear combination of features (output sites or input sites), or weights, comprise it. Input and output counts per brain region were converted to fraction data to account for variation in total number of cells across brains. Fraction data were scaled so that variations in larger regions do not provide oversized contributions to PCA, compared to smaller regions. Analyses were performed in Python using Scikit-learn’s PCA implementation (Pedregosa et al., 2011).

Uniform Manifold Approximation and Projection (UMAP) was used as a non-linear dimensional reduction technique on output and input data. UMAP is better optimized for finding local and global structures in high dimensional data than PCA, but it is far less interpretable. Analyses were performed using the official UMAP library (McInnes et al., 2018). The fractional counts data were z-scored to compare variation in output and input sites across regions with different magnitudes of counts. Z-scored data were dimensionally reduced with UMAP to find clusters of output and input sites with similar patterns of variation. UMAP parameters were tuned manually to optimize stability of clusters.

### Regression Analysis of RABV Input Data

Linear regression was used to quantify the relationship of starter cell COM with the RABV input principal components. Slopes returned from the analysis reflect to what degree lateral and dorsal location increase, decrease, or have no effect on principal components. *p*-values give the probability of these slopes being 0 given the observed data. The statsmodels Python library was used to train these models and examine the slopes (Seabold and Perktold, 2010).

Logistic regression was used to classify the different projection conditions based on the RABV starter cell COMs and the RABV input principal components. To build a model for multiclass classification, we trained a separate logistic regression model to classify each projection condition. When evaluated against a given brain, the model prediction with the highest probability was used. Logistic regression coefficients represent the increased or decreased likelihood of the model prediction given a higher or lower value of a given feature. For example, a positive coefficient for Feature A means the model prediction increases in likelihood for higher values of Feature A and decreases for lower values. A negative coefficient has the opposite relationship; the model prediction increases in likelihood for lower values and decreases for higher values. The higher magnitude of the coefficient, the higher the importance of that feature on the prediction. The Scikit-learn implementation of logistic regression in Python was used for our analysis (Pedregosa et al., 2011).

### Principal Component Analysis of Allen Mouse Brain Connectivity Data

For each of the input regions considered in the RABV experiments, we manually selected corresponding samples from the Allen Mouse Brain Connectivity Data. Experiments were selected based on whether or not the experiment contained labeled projections to the ventral midbrain. NAcLat was not included as an input site, as there were no samples that contained injections that were specific to NAcLat that also projected to the ventral midbrain. Cortex was subdivided into the orbital area and the combined infralimbic and prelimbic areas since our original quantification of RABV inputs included a broad spatial domain not encompassed by any single set of injections. The ID and hyperlink of each sample selected is provided in Supplementary Table 1. For each input region, the sample projections to the VTA were averaged together. Projections were sliced into a 42 pixel x 49 pixel rectangle to capture the largest coronal section of the VTA along with some of the surrounding area. PCA was used to find linear combinations of input regions that maximize variation across the pixels of this rectangle. PC values for each pixel were visualized on the original rectangular space to see how this variation is organized spatially within and around the VTA. These spatial projection “archetypes,” revealed by each principal component’s visualization, were compared to the primary regions that comprise them. Allen samples were accessed using the allensdk python library,^1^ and PCA was performed using Scikit-learn (Pedregosa et al., 2011).

### Allen and RABV Input Clustering Comparison

Clustering of input regions was compared between the RABV input data and the Allen Mouse Brain Connectivity Data (Figures 5C,D,G,H). Z-scoring was performed as before on the input data, capturing variation for each input across the samples. Z-scored data were dimensionally reduced with UMAP to find clusters of inputs with similar variations in each dataset. To account for variability in embeddings, we ran these embeddings 20 times for each dataset and took the average relative pairwise distance between each region. These pairwise distances were computed relative to the maximum distance between any two points in each embedding. Regions were hierarchically clustered based on this distance matrix and compared across datasets.

In order to assess how much clustering we might expect in a random dataset with a similar distribution, we shuffled both RABV and Allen datasets. The z-scored input values were shuffled independently for each region across samples. This eliminated any association between input values for each sample across regions. The clustering comparison analysis was repeated as above.

## Data Availability Statement

All code and data used to complete the analysis and generate the figures in this paper are publicly available in a repository on GitHub: https://github.com/pderdeyn/vtada-network.

## Ethics Statement

The animal study was reviewed and approved by the University of California IACUC and the Stanford University IACUC.

## Author Contributions

PD performed computational analyses. MH made figures. KB organized the results and led manuscript writing and submission. All authors contributed to writing the manuscript.

## Conflict of Interest

The authors declare that the research was conducted in the absence of any commercial or financial relationships that could be construed as a potential conflict of interest.

## Publisher’s Note

All claims expressed in this article are solely those of the authors and do not necessarily represent those of their affiliated organizations, or those of the publisher, the editors and the reviewers. Any product that may be evaluated in this article, or claim that may be made by its manufacturer, is not guaranteed or endorsed by the publisher.

## Footnotes

1
https://github.com/AllenInstitute/AllenSDK

## Supplementary Material

Supplementary Figure 1 VTA^DA^ outputs without the four targeted projection sites. **(A)** Cumulative explained variance from each principal component. **(B)** Samples are plotted in PCA space for the 1st and 2nd components, colored by projection. **(C)** Samples are plotted in PCA space for the 1st and 3rd components, colored by projection. **(D)** Heatmap of each output region’s contribution to the first three principal components. **(E)** Brains are plotted in UMAP space, colored by projection. **(F)** Output regions are plotted in UMAP space, embedded with respect to z-scores across mouse brains. Clusters represent outputs with similar patterns of variation across the cohort.

Supplementary Figure 2 VTA input dimensional reduction without non-Cre and projection-undefined conditions. **(A)** Brains are plotted in PCA space for the 1st and 2nd components, colored by cell type. **(B)** Brains are plotted in PCA space for the 1st and 2nd components, colored by projection. **(C)** Brains are plotted in UMAP space, colored by cell type. **(D)** Brains are plotted in UMAP space, colored by projection.

Supplementary Figure 3 Input and output z-scores stitched together for all cell types. **(A)** Z-scores of average input and output counts for each projection condition. Inputs are marked with a green down arrow and outputs with a red up arrow. Regions are sorted according to the projection in which they receive the highest rank.

Supplementary Figure 4 Revealing RABV input PCs and starter cell locations that predict projection conditions. **(A)** Logistic regression coefficients for five principal components. Positive coefficients predict this condition when the feature is higher. Negative coefficients predict this condition when the feature is lower. Model scores are provided in Table 3. **(B)** Logistic regression coefficients for starter cell location. **(C)** Logistic regression coefficients for five principal components and starter cell location.

Supplementary Figure 5 Projection portraits for all inputs from the Allen Brain Connectivity Atlas. All regions are in the same order as index from Figure 4C.
