# Supplementary figures and images for: Uncovering the Connectivity Logic of the Ventral Tegmental Area
Source: Front Neural Circuits. 2022 Jan 28;15:799688. doi: 10.3389/fncir.2021.799688 (PMC8832514; doi:10.3389/fncir.2021.799688)

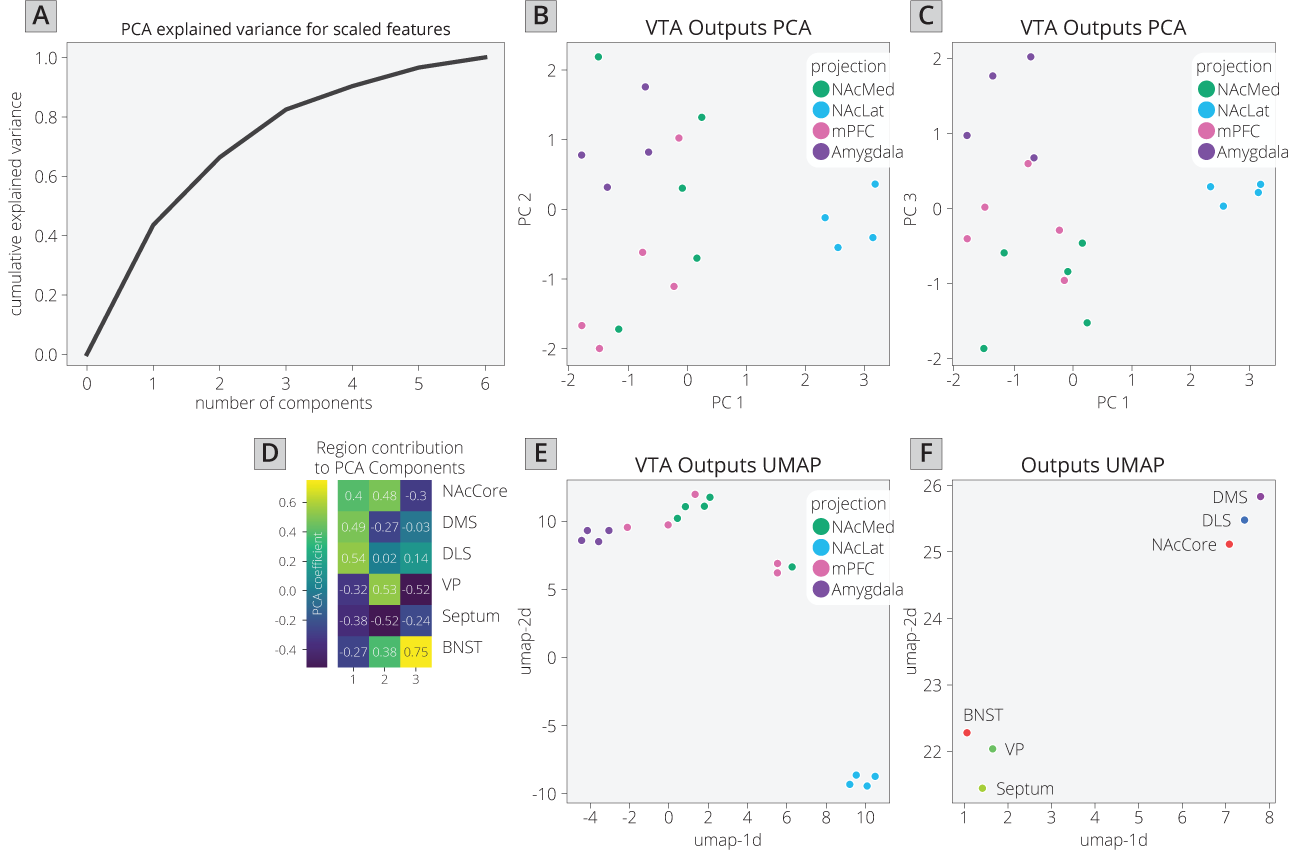

Supplement: Supplementary Figure 1 — VTADA outputs without the four targeted projection sites. (A) Cumulative explained variance from each principal component. (B) Samples are plotted in PCA space for the 1st and 2nd components, colored by projection. (C) Samples are plotted in PCA space for the 1st and 3rd components, colored by projection. (D) Heatmap of each output region’s contribution to the first three principal components. (E) Brains are plotted in UMAP space, colored by projection. (F) Output regions are plotted in UMAP space, embedded with respect to z-scores across mouse brains. Clusters represent outputs with similar patterns of variation across the cohort. [file Image_1.TIF]

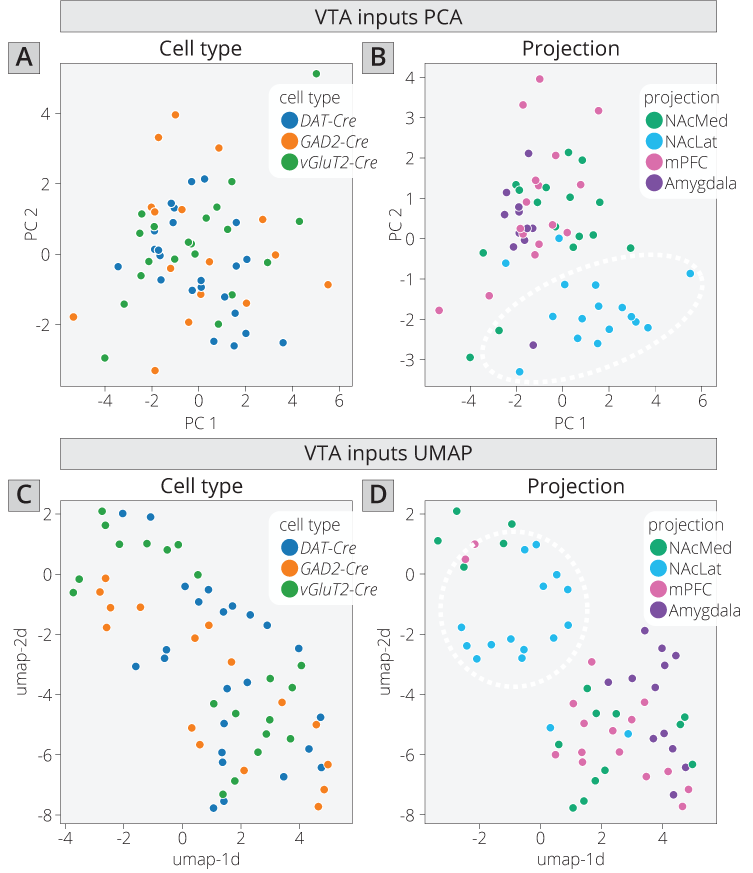

Supplement: Supplementary Figure 2 — VTA input dimensional reduction without non-Cre and projection-undefined conditions. (A) Brains are plotted in PCA space for the 1st and 2nd components, colored by cell type. (B) Brains are plotted in PCA space for the 1st and 2nd components, colored by projection. (C) Brains are plotted in UMAP space, colored by cell type. (D) Brains are plotted in UMAP space, colored by projection. [file Image_2.TIF]

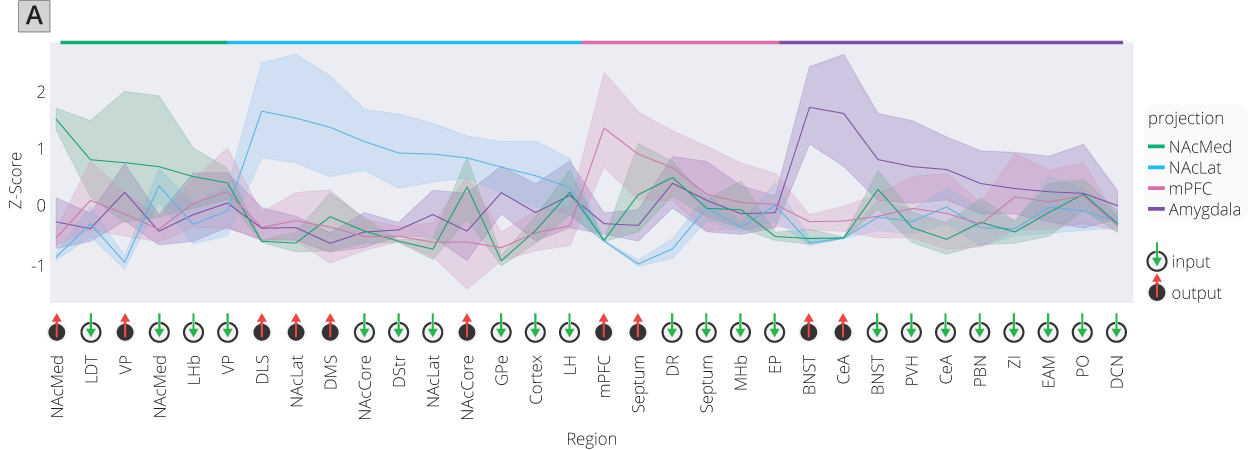

Supplement: Supplementary Figure 3 — Input and output z-scores stitched together for all cell types. (A) Z-scores of average input and output counts for each projection condition. Inputs are marked with a green down arrow and outputs with a red up arrow. Regions are sorted according to the projection in which they receive the highest rank. [file Image_3.TIF]

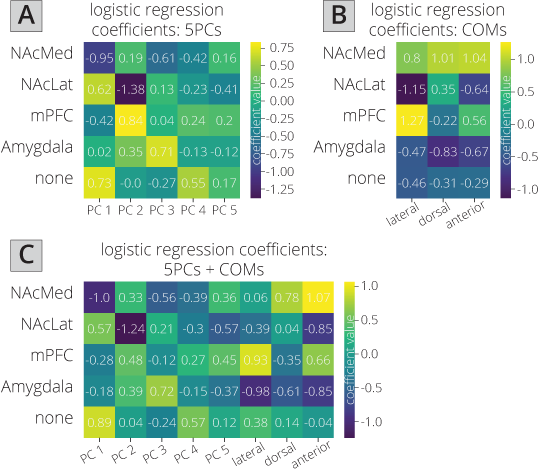

Supplement: Supplementary Figure 4 — Revealing RABV input PCs and starter cell locations that predict projection conditions. (A) Logistic regression coefficients for five principal components. Positive coefficients predict this condition when the feature is higher. Negative coefficients predict this condition when the feature is lower. Model scores are provided in Table 3. (B) Logistic regression coefficients for starter cell location. (C) Logistic regression coefficients for five principal components and starter cell location. [file Image_4.TIF]

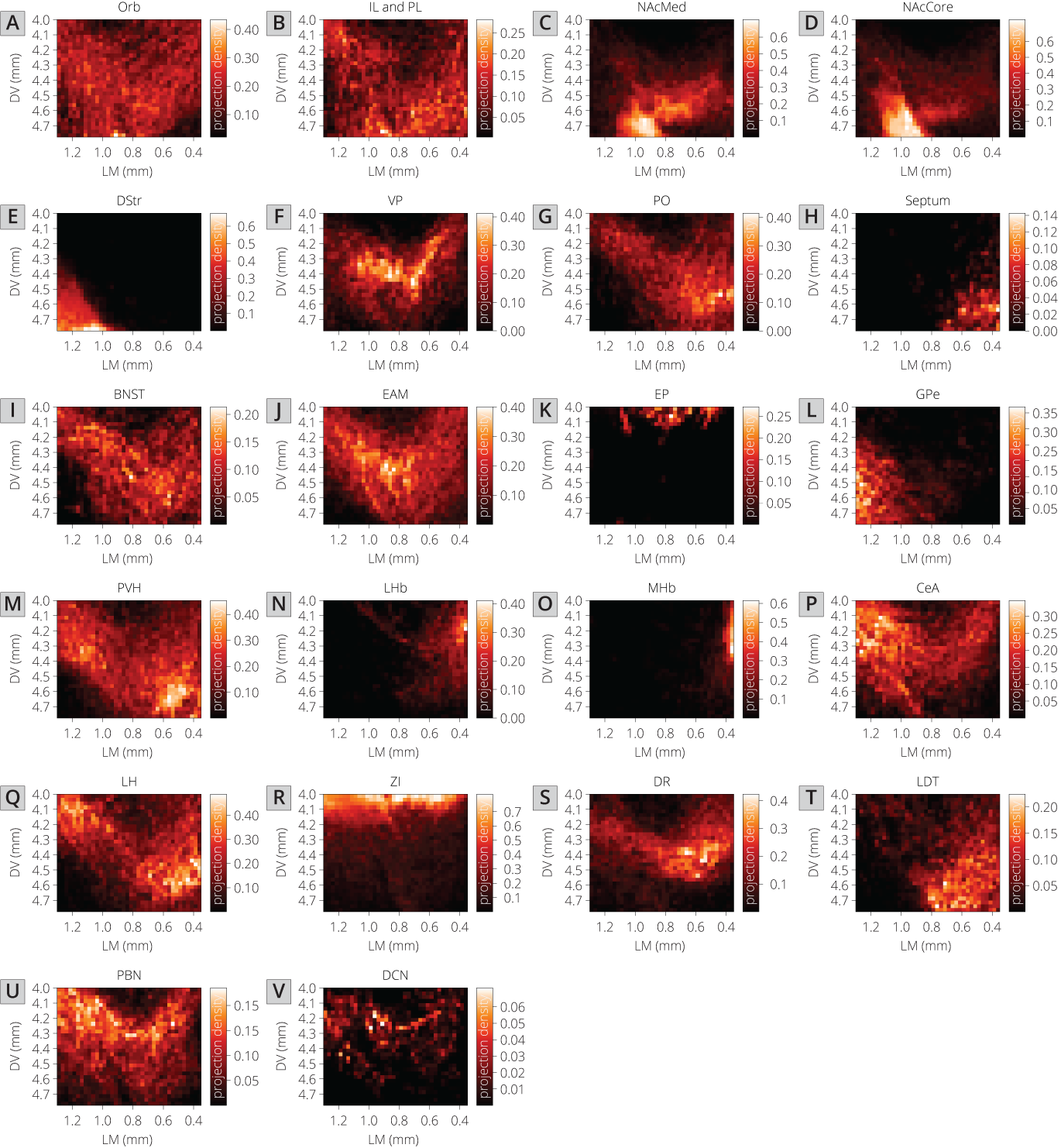

Supplement: Supplementary Figure 5 — Projection portraits for all inputs from the Allen Brain Connectivity Atlas. All regions are in the same order as index from Figure 4C. [file Image_5.TIF]
